# Single-Incision Laparoscopic Hartmann’s Reversal Using the Stoma Site in a Patient with Perioperative Anaphylaxis

**DOI:** 10.70352/scrj.cr.26-0206

**Published:** 2026-07-01

**Authors:** Tsubasa Yanagihara, Ryohei Shoji, Yusuke Yoshida, Nobuhiko Kanaya, Yuki Matsumi, Kunitoshi Shigeyasu, Yoshitaka Kondo, Toshiyoshi Fujiwara

**Affiliations:** Department of Gastroenterological Surgery, Okayama University Graduate School of Medicine, Dentistry and Pharmaceutical Sciences, Okayama, Okayama, Japan

**Keywords:** Hartmann’s reversal, single-incision laparoscopic surgery, parastomal hernia, perioperative anaphylaxis, chlorhexidine allergy

## Abstract

**INTRODUCTION:**

Reversal of Hartmann’s procedure is associated with substantial morbidity, and further reduction of surgical invasiveness may be beneficial in selected patients. Perioperative anaphylaxis caused by anesthetic agents or skin antiseptics poses a significant challenge to surgical management. We report a case of stoma-site single-incision laparoscopic Hartmann’s reversal (HR) performed in a patient with severe perioperative allergies.

**CASE PRESENTATION:**

A 73-year-old woman underwent an emergency open Hartmann’s procedure with partial cystectomy for perforated sigmoid colon cancer with bladder invasion. After adjuvant chemotherapy, she developed a progressively enlarging giant parastomal hernia that significantly impaired daily activities. Two attempts at HR were aborted because of perioperative anaphylactic shock. Subsequent allergy evaluations suggested rocuronium and chlorhexidine as causative agents, and povidone-iodine was also avoided based on clinical history. Approximately 14 months after the initial surgery, single-incision laparoscopic HR using the stoma site was successfully performed under general anesthesia without neuromuscular blocking agents. Preoperative skin antisepsis was performed using benzalkonium chloride, avoiding both chlorhexidine and povidone-iodine. No additional abdominal incisions were required. The operative time was 2 h 56 min, with minimal blood loss. The postoperative course was uneventful and she was discharged on POD 9.

**CONCLUSIONS:**

Stoma-site single-incision laparoscopic HR may represent a useful surgical option for selected patients with severe perioperative allergies to anesthetic agents and skin antiseptics, in whom minimizing abdominal approach and wound-related risks is particularly important.

## Abbreviations

HR
: Hartmann’s reversal
NMBAs
: neuromuscular blocking agents
SSI
: surgical site infection

## INTRODUCTION

Hartmann’s procedure is frequently performed in emergency settings for complicated colorectal diseases, including perforated colorectal cancer and diverticulitis. In particular, patients with perforated sigmoid colon cancer often require an open Hartmann’s procedure because of the risks of severe peritoneal contamination and physiological instability. Although this approach is lifesaving, HR remains technically demanding and is associated with substantial morbidity, such as SSI, postoperative ileus, and prolonged hospital stay.^1)^

To reduce surgical invasiveness, laparoscopic HR has been increasingly adopted and has been shown to be feasible and safe in selected patients. Previous studies have reported advantages of the laparoscopic approach over open surgery, including reduced blood loss and shorter hospital stay.^1,2)^ More recently, single-incision or single-port laparoscopic techniques using the stoma site as the sole access point have been introduced to further minimize abdominal wall trauma and avoid additional incisions. Several reports have demonstrated the technical feasibility and acceptable short-term outcomes of stoma-site single-incision HR, suggesting that this approach may represent a viable minimally invasive option in selected cases.^3,4)^

Perioperative anaphylaxis is a rare but potentially life-threatening complication, most commonly caused by NMBAs and antiseptic agents such as chlorhexidine.^5,6)^ Current recommendations emphasize strict avoidance of suspected causative agents identified through allergy evaluation and careful selection of alternative perioperative drugs and antiseptic strategies for subsequent surgical procedures.^5)^ However, patients who develop hypersensitivity reactions to multiple perioperative agents provide a particularly challenging clinical scenario, particularly with regard to infection prevention and wound management.

We report herein a case of stoma-site single-incision laparoscopic HR performed in a patient with a history of perioperative anaphylaxis to NMBAs and preoperative skin antiseptics. This case highlights the feasibility of a stoma-site single-incision approach as a surgical option for patients in whom perioperative medications and antiseptic choices are severely limited.

## CASE PRESENTATION

A 73-year-old woman was referred to our department for stoma-related complications. She had previously been diagnosed with sigmoid colon cancer with bladder invasion and initially received 1 course of neoadjuvant chemotherapy with 5-fluorouracil, leucovorin, oxaliplatin, and irinotecan plus bevacizumab. She subsequently developed tumor perforation and underwent an emergency open Hartmann’s procedure with D3 lymphadenectomy and partial cystectomy.^7)^ For this surgery, olanexidine was used as an antiseptic and rocuronium was used as a muscle relaxant. Histopathological examination revealed a moderately differentiated tubular adenocarcinoma measuring 45 × 40 mm, staged as pT4b (bladder), N0, M0, stage II c.^7)^ The patient subsequently completed 8 courses of adjuvant capecitabine plus oxaliplatin chemotherapy, and follow-up CT showed no evidence of tumor recurrence or distant metastasis.

Several months after the initial surgery, the patient developed a progressively enlarging parastomal hernia. Preoperative CT revealed a giant parastomal hernia with protrusion of the small intestine through the abdominal wall defect at the stoma site. The size of the hernia orifice was 60 × 50 mm, corresponding to Type III in the European Hernia Society classification of parastomal hernias^8)^ (**Fig. 1**). As the stoma site became increasingly deformed, making it difficult to secure a stable pouch, she strongly requested stoma closure and was referred to our department approximately 11 months after the initial Hartmann’s procedure.

**Fig. 1:**
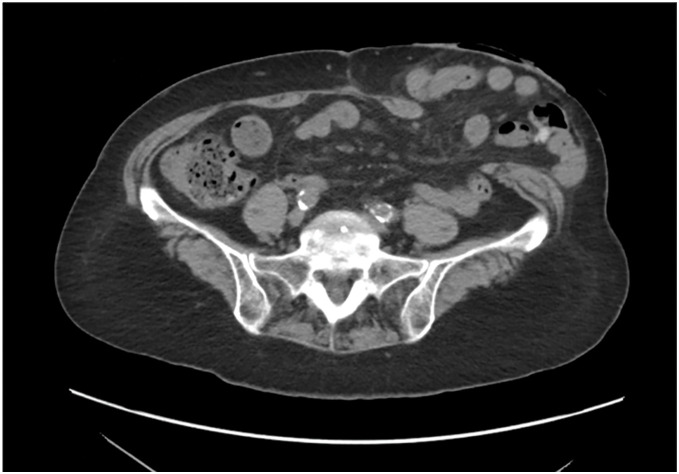
Preoperative abdominal CT showing a giant parastomal hernia with protrusion of the intestinal tract through the abdominal wall at the stoma site.

At the first attempt at HR, the patient developed anaphylactic shock during the induction of general anesthesia, leading to cancellation of the procedure. Subsequent dermatological evaluations with skin prick testing identified rocuronium as the suspected causative agent. Several months later, a second attempt at surgery was planned to convert to a laparotomy with complete avoidance of NMBAs; instead, an epidural anesthetic was added, but the patient again experienced anaphylactic shock during general anesthesia, and the operation was aborted. Further allergy testing suggested hypersensitivity to chlorhexidine, which had been used to disinfect the skin prior to intravenous cannulation and an epidural puncture. Additionally, povidone-iodine, which had been used to antiseptically treat the surgical site immediately before the onset of allergic symptoms, was also considered a suspected causative agent.

Weighing the severely limited perioperative medication and antiseptic options against the worsening parastomal hernia, a surgical strategy was carefully planned to minimize additional surgical trauma. Approximately 14 months after the initial Hartmann’s procedure, the patient underwent single-incision laparoscopic HR using the stoma site under general anesthesia without NMBAs. General anesthesia with sevoflurane, propofol, and remifentanil was supplemented with epidural anesthesia using ropivacaine and spinal anesthesia using isobaric bupivacaine. Preoperative skin antisepsis was performed using benzalkonium chloride, avoiding chlorhexidine, olanexidine, and povidone-iodine. The patient was placed in the lithotomy position. Primarily, the colostomy was mobilized and freed from the fascia and hernia sac. The free stump of the colostomy was temporarily closed by suturing. The lifted colon was returned into the abdominal cavity. Any adhesions close to the wound in the abdominal cavity under direct visualization were freed. The ALEXIS O-Ring Wound Retractor (Applied Medical, Rancho Santa Margarita, CA, USA) and Free Access (S) (Top, Tokyo, Japan) were then placed as the single-port access platform in the fascial defect at the colostomy site, and pneumoperitoneum of 10 mmHg was established. A flexible laparoscope with a 5-mm-diameter tip, ENDOEYE FLEX HD 5 mm (Olympus Medical Systems, Tokyo, Japan), was introduced. The patient was subsequently positioned in the anti-Trendelenburg position, making the small pelvis visible. Intra-abdominal procedures were performed using 4 EZ Trocar Smart Insertion 5 mm (Hakko, Nagano, Japan) (**Fig. 2A**). The detachment of adhesions formed at the previous midline incision was minimized; adhesions surrounding the rectal stump were extensively dissected by either sharp dissection with laparoscopic scissors or HARMONIC 1100 (Ethicon, Raritan, NJ, USA) until the rectal stump (**Fig. 2B**). We confirmed that tension-free anastomosis could be performed without mobilizing the splenic flexure of the colon. After temporarily ceasing insufflation, the anvil of the ECHELON Circular Powered Stapler CDH25 (Ethicon) was secured in the proximal colon using a purse-string suture under direct visualization. Under repeat pneumoperitoneum, the circular stapler was advanced via the anus, and an end-to-side anastomosis was performed within the abdominal cavity (**Fig. 3A**). The pneumoperitoneum was released and the platform was removed after placing the 19-Fr Blake silicone drain (Ethicon) from the right lower abdomen to the anastomosis site. Finally, the hernia sac was excised under direct visualization and the fascia was closed with interrupted sutures (**Fig. 3B**). The operation time was 2 h 56 min, with minimal blood loss. No intraoperative complications related to the change in anesthesia technique were observed. The postoperative course was uneventful. No SSI or other postoperative complications were observed. Oral intake was resumed without difficulty and the patient was discharged on POD 9 in good condition.

**Fig. 2:**
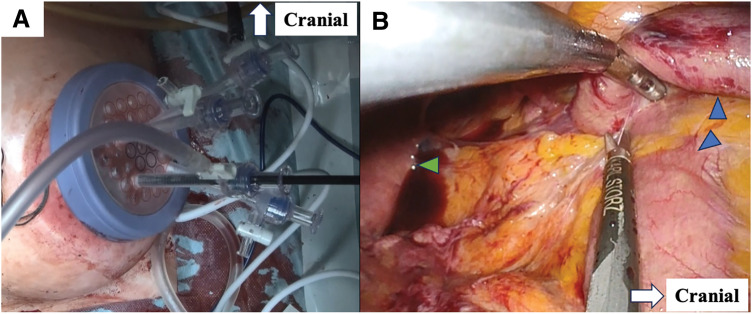
Intraoperative findings during single-incision laparoscopic HR using the stoma site. (**A**) A single-port access device was placed at the stoma site, allowing laparoscopic surgery without additional abdominal incisions. (**B**) Adhesiolysis in the pelvic cavity was successfully performed under a single-incision laparoscopic approach. Blue arrowheads indicate the small intestine. The green arrowhead indicates the uterus. HR, Hartmann’s reversal

**Fig. 3:**
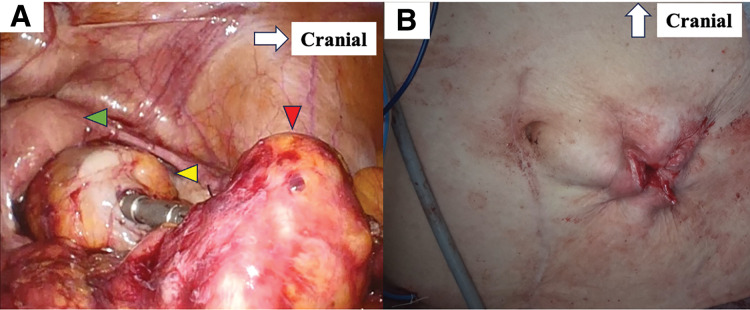
Intraoperative and postoperative findings of single-incision laparoscopic HR. (**A**) Intracorporeal colorectal anastomosis performed under a single-incision laparoscopic approach. The red arrowhead indicates the proximal colon. The yellow arrowhead indicates the rectal stump. The green arrowhead indicates the uterus. (**B**) Postoperative abdominal view demonstrating completion of the procedure using the stoma site without additional abdominal incisions. HR, Hartmann’s reversal

## DISCUSSION

In the present case, surgical management was complicated by repeated episodes of perioperative anaphylaxis.

The use of NMBAs is generally essential for securing an adequate operative field during pneumoperitoneum.^9)^ Although brief laparoscopic procedures without NMBAs have been reported to be feasible in gynecologic surgery,^10)^ this strategy may not be suitable for HR cases in which extensive intra-abdominal adhesions are anticipated preoperatively. In such cases, surgeons must consider the risks associated with trocar placement at multiple lateral abdominal wall sites, the technical difficulty of adhesiolysis—which is a major cause of conversion to laparotomy^1)^—and the potential for prolonged operative time. Under these circumstances, it is questionable whether a multi-port laparoscopic approach should be the first-line option when adequate intra-abdominal working space cannot be reliably secured. Moreover, these anesthetic limitations may also compromise visualization and operative exposure during open surgery.

On the other hand, SSI is the most common postoperative complication following HR. Given the reported advantages of laparoscopic surgery over open surgery with respect to SSI prevention^2)^, a laparoscopic approach was considered particularly beneficial in the present case, in which standard preoperative skin antisepsis should ideally be avoided.^11)^

Single-incision laparoscopic HR through the stoma site has been reported as a feasible and minimally invasive technique in selected patients.^3,4,12)^ By establishing a single access platform with multiple trocars through the stoma site at the beginning of the procedure, abdominal entry can be achieved safely without the need for additional lateral abdominal wall trocars. Furthermore, approaching the rectal stump directly—the principal target of this operation—may reduce the need for adhesiolysis beneath the previous midline incision, which is often required in open or conventional multi-port laparoscopic procedures. This technique may also avoid additional abdominal incisions and thereby reduce incision-related infectious complications. Previous reports have demonstrated an average conversion rate to open surgery of approximately 12% for conventional multi-port procedures, whereas single-incision procedures showed a 4% conversion rate to open surgery and an 8% rate of additional port placement.^12)^ Although direct comparison is difficult, these findings support the potential rationale for a single-incision approach in HR. In the present case, the fascial defect associated with the parastomal hernia allowed placement of a relatively large single-port access platform, providing sufficient distance between trocars. This reduced instrument interference—a common technical limitation of single-incision laparoscopic surgery^13)^—and facilitated safe intra-abdominal manipulation.

Several limitations should be acknowledged. This report describes a single case, and the findings therefore cannot be generalized. In addition, the long-term outcomes of stoma-site single-incision HR in patients with perioperative allergies remain unclear. Further accumulation of cases is necessary to clarify the safety, reproducibility, and indications of this approach.

## CONCLUSIONS

Single-incision laparoscopic HR using the stoma site proved feasible in a patient with a history of anaphylaxis to NMBAs and preoperative antiseptics.

## DECLARATIONS

### Availability of data and materials

The data that support the findings of this study are available from the corresponding author upon reasonable request.
